# Functional impairment of Tax-specific but not cytomegalovirus-specific CD8^+ ^T lymphocytes in a minor population of asymptomatic human T-cell leukemia virus type 1-carriers

**DOI:** 10.1186/1742-4690-8-100

**Published:** 2011-12-07

**Authors:** Ayako Takamori, Atsuhiko Hasegawa, Atae Utsunomiya, Yasuhiro Maeda, Yoshihisa Yamano, Masato Masuda, Yukiko Shimizu, Yotaro Tamai, Amane Sasada, Na Zeng, Ilseung Choi, Naokuni Uike, Jun Okamura, Toshiki Watanabe, Takao Masuda, Mari Kannagi

**Affiliations:** 1 Department of Immunotherapeutics, Tokyo Medical and Dental University, Tokyo, Japan; 2 Department of Hematology, Imamura Bun-in Hospital, Kagoshima, Japan; 3 Division of Hematology, Department of Internal Medicine, Kinki University School of Medicine, Osaka, Japan; 4 Department of Molecular Medical Science, Institute of Medical Science, St. Marianna University School of Medicine, Kawasaki, Japan; 5 Cancer Centre, University of the Ryukyus Hospital, Okinawa, Japan; 6 Department of Hematology, National Kyushu Cancer Center, Fukuoka, Japan; 7 Institute for Clinical Research, National Kyushu Cancer Center, Fukuoka, Japan; 8 Laboratory of Tumor Cell Biology, Department of Medical Genome Science, Graduate School of Frontier Sciences, The University of Tokyo, Tokyo, Japan; 9 Department of Hematology, Osaka Minami Medical Center, Osaka, Japan

## Abstract

**Background:**

Human T-cell leukemia virus type 1 (HTLV-1) causes adult T-cell leukemia (ATL) and HTLV-1-associated myelopathy/tropical spastic paraparesis (HAM/TSP) in a small percentage of infected individuals. ATL is often associated with general immune suppression and an impaired HTLV-1-specific T-cell response, an important host defense system. We previously found that a small fraction of asymptomatic HTLV-1-carriers (AC) already showed impaired T-cell responses against the major target antigen, Tax. However, it is unclear whether the impaired HTLV-1 Tax-specific T-cell response in these individuals is an HTLV-1-specific phenomenon, or merely reflects general immune suppression. In this study, in order to characterize the impaired HTLV-1-specific T-cell response, we investigated the function of Tax-specific CD8^+ ^T-cells in various clinical status of HTLV-1 infection.

**Results:**

By using tetramers consisting of HLA-A*0201, -A*2402, or -A*1101, and corresponding Tax epitope peptides, we detected Tax-specific CD8^+ ^T-cells in the peripheral blood from 87.0% of ACs (n = 20/23) and 100% of HAM/TSP patients (n = 18/18) tested. We also detected Tax-specific CD8^+ ^T-cells in 38.1% of chronic type ATL (cATL) patients (n = 8/21), although its frequencies in peripheral blood CD8^+ ^T cells were significantly lower than those of ACs or HAM/TSP patients. Tax-specific CD8^+ ^T-cells detected in HAM/TSP patients proliferated well in culture and produced IFN-γ when stimulated with Tax peptides. However, such functions were severely impaired in the Tax-specific CD8^+ ^T-cells detected in cATL patients. In ACs, the responses of Tax-specific CD8^+ ^T-cells were retained in most cases. However, we found one AC sample whose Tax-specific CD8^+ ^T-cells hardly produced IFN-γ, and failed to proliferate and express activation (CD69) and degranulation (CD107a) markers in response to Tax peptide. Importantly, the same AC sample contained cytomegalovirus (CMV) pp65-specific CD8^+ ^T-cells that possessed functions upon CMV pp65 peptide stimulation. We further examined additional samples of two smoldering type ATL patients and found that they also showed dysfunctions of Tax-specific but not CMV-specific CD8^+ ^T-cells.

**Conclusions:**

These findings indicated that Tax-specific CD8^+ ^T-cells were scarce and dysfunctional not only in ATL patients but also in a limited AC population, and that the dysfunction was selective for HTLV-1-specifc CD8^+ ^T-cells in early stages.

## Background

Human T-cells leukemia virus type 1 (HTLV-1) is the causative agent of a highly aggressive CD4^+ ^T-cell malignancy, adult T-cell leukemia (ATL)[1,2]. As many as 10 million individuals are thought to be infected worldwide, in southern Japan, the Caribbean basin, South America, Melanesia, and equatorial Africa[3]. Unlike human immunodeficiency virus (HIV), the majority of HTLV-1-infected individuals are clinically asymptomatic during their lifetime. However, approximately 5% develop ATL, and another 2-3% develop a variety of chronic inflammatory diseases such as HTLV-1-associated myelopathy/tropical spastic paraparesis (HAM/TSP)[4-8].

HTLV-1-specific cytotoxic T-lymphocytes (CTLs) are thought to play a pivotal role in containing the proliferation of HTLV-1-infected T-cells[9,10]. Tax is known to be the dominant target antigen for HTLV-1-specific CTLs[10-13], and a high frequency of Tax-specific CTLs can be detected in HAM/TSP patients and some asymptomatic HTLV-1 carriers (ACs)[10-14]. However, ATL patients show general immune suppression[15], reduced frequency and dysfunction of Tax-specific CTLs[16,17]. Regulatory T cell (Treg)-like function of FoxP3^+ ^ATL cells and diminished function of dendritic cells may be involved in the immune suppression in ATL patients[18,19], but the precise mechanism is not yet clarified. We previously demonstrated that a fraction of ACs also exhibit reduced T-cell responses against Tax protein[20]. These observations suggest that the reduced HTLV-1-specific T-cell response might be an underlying risk of ATL development, but not the result of ATL. However, it is unknown how the function of HTLV-1-specific CD8^+ ^T-cells becomes impaired in a small percentage of ACs and whether its dysfunction is specific for HTLV-1 antigen or due to general immune suppression.

During chronic stage of infection with several viruses, such as HIV and hepatitis C virus (HCV), virus-specific CTLs gradually lose their cytotoxic activity, the ability to proliferate and secrete a diverse profile of cytokines, ultimately leading to exhaustion, anergy or even deletion of these cells[21-26]. Programmed death-1 (PD-1), a negative regulator in the CD28 superfamily, has recently been shown to be highly expressed on virus-specific T-cells during many chronic viral infections[27-29]. It has also been reported that the interaction of PD-1 with PD-ligand 1 (PD-L1) negatively regulates cytokine production and proliferation of T-cells[30,31]. A previous report indicates that PD-1 is up-regulated on the dominant Tax-specific CTLs in ATL patients and ACs and that immune regulation through the PD-1/PD-L1 pathway may be involved in the dysfunction of HTLV-1-specific CTLs in ATL patients[32].

Studies on memory T-cell differentiation have shown that phenotype, function, and homeostasis of memory T-cells vary for different persistent virus infections[33]. Central memory T-cells (T_CM_; CD45RA^-^CCR7^+^) are elicited by non-persisting virus that provide transient antigen stimulation, such as in Influenza virus infection. In contrast, effector memory T-cells (T_EM_; CD45RA^-^CCR7^-^) predominate when relatively high levels of antigen persist, such as in HIV infection. Terminally differentiated memory (T_Diff_; CD45RA^+^CCR7^-^) can be seen when antigen persists at a low level, such as in cytomegalovirus (CMV) infection. In HTLV-1 infection, it has been reported that dominant Tax-specific CTLs in HAM/TSP patients consist of T_EM _and T_Diff _compartments[34].

We previously identified some major epitopes recognized by HTLV-1-specific CTLs in infected individuals carrying HLA-A2, -A11, or -A24[12,35,36]. These allowed us to monitor HTLV-1-specific CTLs and analyze their functions *ex vivo*, by using antigen/HLA tetrameric complexes. In this study, we demonstrate that IFN-γ production and proliferative capacity of tetramer-binding Tax-specific CD8^+ ^T-cells were severely impaired not only in ATL patients but also in a minor population of asymptomatic HTLV-1 carriers (ACs). Importantly, the T-cell dysfunction at the asymptomatic stage was selective for HTLV-1 but not for CMV antigen. In addition, severely impaired HTLV-1-specific but not CMV-specific CD8^+ ^T-cells responses were also observed in patients diagnosed as smoldering ATL, the clinical condition of which is close to that of AC. The dysfunction of HTLV-1-specific CD8^+ ^T-cells in an early clinical stage implies HTLV-1-specific immune suppressive mechanism might be an underlying risk for ATL.

## Results

### Incidence and frequency of Tax-specific CD8^+ ^T-cells in ACs, and HAM/TSP and cATL patients

**Figure 1:**
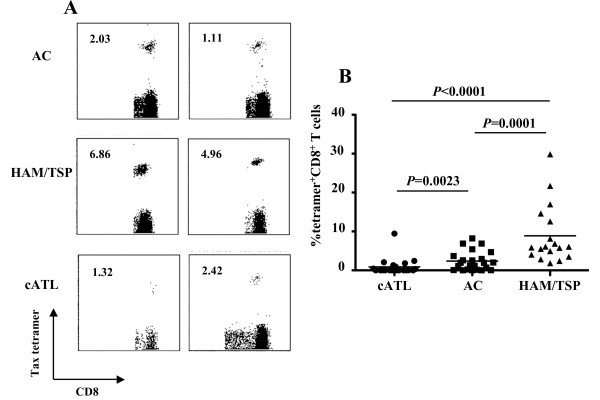
**Incidence and frequency of Tax-specific CD8^+ ^T-cells in ACs, and HAM/TSP and cATL patients**. (A) Whole blood or PBMCs from AC (top), and HAM/TSP (middle) and cATL (bottom) patients were stained with Tax/HLA tetramer. Number indicates the percentage of tetramer^+ ^cells in CD8^+ ^T-cells. (B) The percentage of Tax-tetramer^+ ^CD8^+ ^T-cells in AC (n = 23), and HAM/TSP (n = 18) and cATL (n = 21) patients. *P *value was determined by the Mann-Whitney *U *test. Horizontal bars indicate the average percentage of Tax-tetramer^+ ^CD8^+ ^T-cells for the group.

In 23 ACs and 18 HAM/TSP and 21 cATL patients carrying HLA-A2, -A11 and/or -A24 alleles, we evaluated the frequencies of Tax-specific CD8^+ ^T-cells by using cognate Tax/HLA tetramers (Figure 1 and Table 1). Tax-specific CD8^+ ^T-cells were detected in 87.0% of ACs and all HAM/TSP patients tested. In contrast, only 38.1% of cATL patients have detectable frequencies of Tax-specific CD8^+ ^T-cells (Table 1). Figure 1B shows that the average frequency of Tax-specific CD8^+ ^T-cells in the CD8^+ ^T-cells of cATL patients (n = 21, 0.90% range: 0%-9.45%) was significantly lower than that in ACs (n = 23, 2.37%, range: 0%-8.23%, *P *= 0.0023). HAM/TSP patients had the highest average frequency of Tax-specific CD8^+ ^T-cells among the three groups (n = 18, 8.88%, range: 1.86%-29.9%, *P *= 0.0001; vs. AC, *P *< 0.0001; vs. cATL patients), which is consistent with previous reports [10,17,37]. It is of note that Tax-specific CD8^+ ^T-cells are detectable even in cATL patients, although the frequency is very low.

**Table 1:** The number of blood samples with detectable Tax-specific CD8^+ ^T-cells in all samples tested in this study

Tax/HLA tetramers used in this study	Disease Status		
	
	AC	HAM/TSP	cATL
HLA-A*0201/Tax11-19	12/14^1^	7/7	2/11
HLA-A*1101/Tax88-96	4/4	4/4	3/5
HLA-A*2402/Tax301-309	13/15	13/13	5/16

No. of tetramer^+ ^samples/total no. of blood samples^2^	20/23 (87.0%)	18/18 (100%)	8/21 (38.1%)

^1 ^No. of samples with detectable Tax-specific CD8^+ ^T-cells/total no. of samples carrying each HLA allele. When the frequency of tetramer^+ ^cells was more than 0.04% of CD8^+ ^T-cells, the sample was regarded as detectable.

^2 ^In case Tax-specific CD8^+ ^T-cells was detectable by either tetramer in a sample carrying two of three HLA-A alleles above, the sample was regarded as positive.

### Impaired cell proliferation and IFN-γ production of Tax-specific CD8^+ ^T-cells in cATL but not HAM/TSP patients

**Figure 2:**
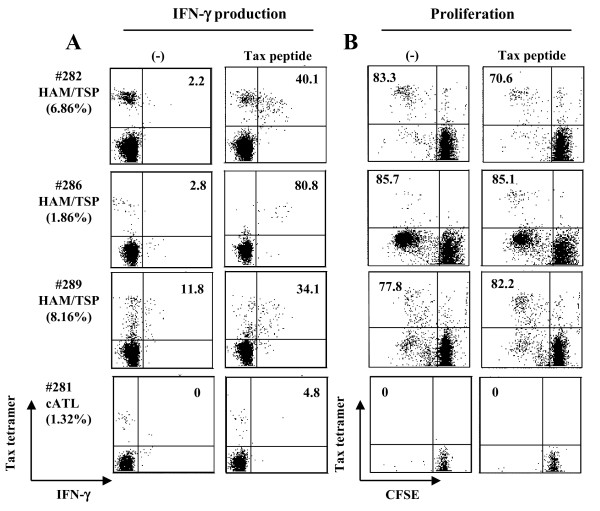
**IFN-γ production and proliferation of Tax-specific CD8^+ ^T-cells in HAM/TSP and cATL patients**. (A) PBMCs from HAM/TSP and cATL patients were stimulated with or without 10 μM Tax peptide for 6 hrs. The number indicates the percentage of IFN-γ-producing cells in tetramer^+ ^cells. (B) For CFSE-based T-cell proliferation, CFSE-labeled PBMCs from HAM/TSP and cATL patients were cultured in the presence or absence of 100 nM Tax peptide for 6 days. The number indicates the percentage of dividing (CFSE^low^) cells in tetramer^+ ^cells. The percentage of tetramer^+ ^cells among CD8^+ ^T cells in fresh blood is indicated in parenthesis under the patient ID.

We next examined IFN-γ production and cell proliferation of Tax-specific CD8^+ ^T-cells in HAM/TSP and cATL patients (Figure 2A). Intracellular IFN-γ staining showed that Tax-specific CD8^+ ^T-cells in all HAM/TSP patients tested produced IFN-γ when stimulated with Tax peptide (Figure 2A). Tax-specific CD8^+ ^T-cells in those HAM/TSP patients proliferated regardless of stimulation with Tax peptide (Figure 2B). In contrast to HAM/TSP patients, IFN-γ production from Tax-specific CD8^+ ^T-cells in a cATL patient was hardly detectable even when stimulated with Tax peptide (4.8%, Figure 2A). In the same donor, Tax-specific CD8^+ ^T-cells could be detected in fresh blood (1.32%) and after 6 hrs incubation as shown in Figure 2A, but not after 6 day-culture, suggesting that Tax-specific CD8^+ ^T-cells in this cATL patient had no proliferative capacity (Figure 2B). We tested PBMC from four other cATL patients who had detectable Tax-specific CD8^+ ^T-cells, but none of them showed proliferation of Tax-specific CD8^+ ^T-cells by either the CFSE-based proliferation assay or 13-day culture (Additional file 1). Collectively, these results indicate that Tax-specific CD8^+ ^T-cells from most cATL patients are impaired in their capacities to proliferate and produce IFN-γ.

### Diversity in the IFN-γ production and cell proliferation of Tax-specific CD8^+ ^T-cells in ACs

**Figure 3:**
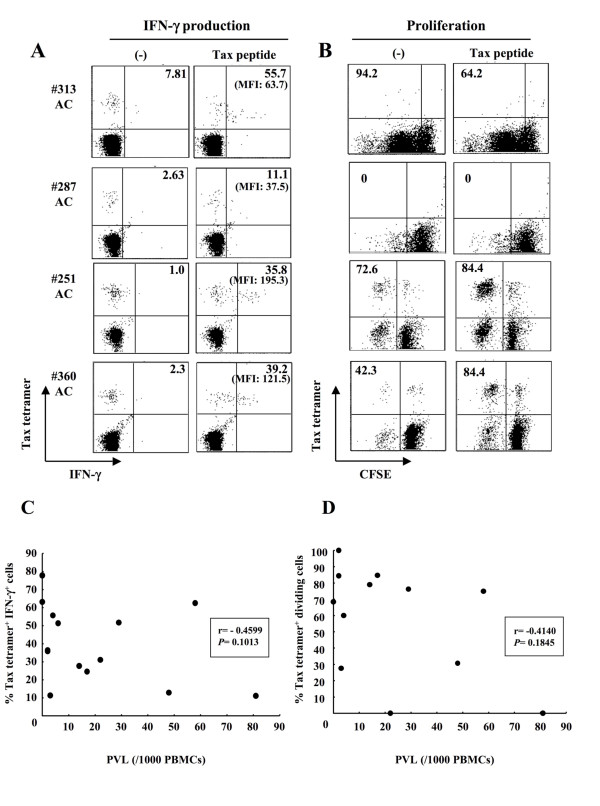
**IFN-γ production and cell proliferation of Tax-specific CD8^+ ^T-cells in ACs**. (A, B) IFN-γ production (A) and cell proliferation (B) of Tax-specific CD8^+ ^T-cells in PBMCs from 4 ACs were assessed as in Figure 2. The number given in parenthesis shows mean fluorescence intensity (MFI) of IFN-γ expression in the IFN-γ^+ ^tetramer^+ ^cells. (C, D) Relation between the percentage of IFN-γ^+ ^(C) or dividing (D) Tax-specific CD8^+ ^T-cells and proviral loads (PVL) in ACs. Dots represent individual ACs. The Spearman rank correlation test was used to determine correlations and *P *values.

Our recent studies using the GST-Tax protein-based assay demonstrated that the extent of Tax-specific T-cell responses varied widely in ACs[20]. We then evaluated proliferation and/or IFN-γ production of tetramer-binding Tax-specific CD8^+ ^T-cells in 14 ACs (Table 2). Representative data on 4 of 14 ACs are shown in Figures 3A and 3B. In 3 ACs (#251, #313, and #360), Tax-specific CD8^+ ^T-cells produced IFN-γ and proliferated in response to Tax peptide (Figures 3A and 3B). Similarly to HAM/TSP samples, a large proportion of Tax-specific CD8^+ ^T-cells in these ACs spontaneously proliferated without stimulation with Tax peptide, probably due to viral reactivation in HTLV-1-infected cells *in vitro*[38,39]. IFN-γ production was specifically detected for peptide stimulation, and 35.8-55.7% of Tax-specific CD8^+ ^T-cells produced a good amount of IFN-γ (mean fluorescence intensity, MFI: 63.7-195.3) upon stimulation in the samples of #251, #313, and #360. In contrast, Tax-specific CD8^+ ^T-cells in one AC (#287) did not proliferate in response to Tax peptide and showed a very weak IFN-γ response with low amounts of IFN-γ (MFI: 37.5) in a low percentage (11.1%) of Tax-specific CD8^+ ^T-cells (Figures 3A and 3B). In other ACs (#243 and #279), low frequency of IFN-γ^+ ^Tax-specific CD8^+ ^T-cells was observed, but the levels of IFN-γ production (MFI: #243; 58.8, #279; 77.6) and the proliferative responses were comparable to other ACs (Table 2). Tax-specific CD8^+ ^T-cells in #236 failed to proliferate but showed favorable IFN-γ production (MFI: 80.1) in 31.1% of the cells.

**Table 2:** Clinical information and summary for Tax-specific CD8^+ ^T cells in 14ACs

ID	Age	Sex	WBC (/μl)	CD4 (%)^1^	CD8 (%)^1^	HLA	Tetramer (%)^2^	Functions and phenotype of Tax-specific CD8^+ ^T-cells^3^			Ably (%)^7^	PVL^8^
										
								IFN-γ^+^ (%)^4^	CFSE^low^ (%)^5^	PD-1^+^ (%)^6^		
#217	70s	F	6800	ND^9^	5.72	A24	1.94	27.7	78.9	78.7	0	14
#236	30 s	F	6500	ND	11.9	A24	2.54	31.1	0	54.1	0	22
#238	60 s	F	5700	ND	12.7	A11	1.29	36.4	100	0	0	2
#243	50 s	F	4100	ND	24.6	A2/24	0.39/3.67	11.3	27.6	93.8	0	3
#245	40 s	F	5000	ND	22.6	A2	0.73	62.5	75	ND	1	58
#251	60 s	M	4800	ND	11.9	A2/11	0.70/8.23	35.8	84.4	36.7	0	2
#279	40 s	M	6200	34.1	11.6	A2/24	4.70/0.18	12.9	30.8	70.2	1	48
#287	70 s	M	4800	72.5	10.0	A2/24	1.17/0.23	11.1	0	55.6	2	81
#309	60 s	F	4600	37.5	24.8	A11/24	6.88/4.26	51.7	76.2	85.3	1.5	29
#311	60 s	F	3200	30.6	14.8	A2/24	1.02/1.94	51.3	ND	ND	0	6
#312	50 s	F	2700	27.3	36.4	A24	2.03	77.8	ND	ND	ND	UN^10^
#313	60 s	M	7300	25.4	31.0	A24	1.11	55.7	60	90.6	ND	4
#315	50 s	F	7500	26.5	7.9	A2/24	6.88/0	24.5	84.7	20	0.6	17
#360	50 s	M	6200	37.7	29.9	A2	2.6	63.1	68.4	10.2	0	UN

^1^The number indicates percentage of CD4^+ ^or CD8^+ ^T cells in lymphocytes.

^2^The number indicates percentages of tetramer^+ ^cells in CD8^+ ^T-cells. Two numbers divided by a slash represent those detected by two different tetramers corresponding to two HLA alleles shown in the HLA column.

^3^In case of a sample carrying two of three HLA-A alleles (A2, A11, or A24), Tax-specific CTLs predominantly detected by a tetramer were used. The number represents percentage of indicated cells in the tetramer-binding CD8^+ ^T cells.

^4^Evaluated by intracellular IFN-γ staining following 6 hours stimulation with corresponding Tax peptide.

^5^Evaluated by CFSE intensities in labeled PBMC after 6 days incubation with corresponding Tax peptide stimulation.

^6^The number represents percentage of indicated PD-1^+^Tax-specific CD8^+ ^T cells without culture.

^7^Ably; abnormal lymphocytes

^8^PVL; proviral load. The number represents copy number per 1000 PBMCs.

^9^ND; not determined

^10^UN; undetectable

Among AC samples tested, AC#287 carried higher proviral load (81 copies in 1000 PBMCs) than any other ACs (Table 2). Since Tax-specific CD8^+ ^T-cells in #287 had severely impaired IFN-γ production and proliferative potential, we examined the relationship of the function of these T-cells with proviral loads. Both percentages of IFN-γ^+ ^and dividing Tax-specific CD8^+ ^T-cells among CD8^+ ^T-cells were likely to be inversely correlated with proviral loads although they were not statistically significant (Figure 3C and 3D). Because of the limited availability of the samples, we focused mainly on two ACs (#287 and #313) in the studies hereafter.

### Dysfunction of Tax-specific CD8^+ ^T-cells and inefficient CD8^+ ^cell-mediated HTLV-1 control in AC #287

**Figure 4:**
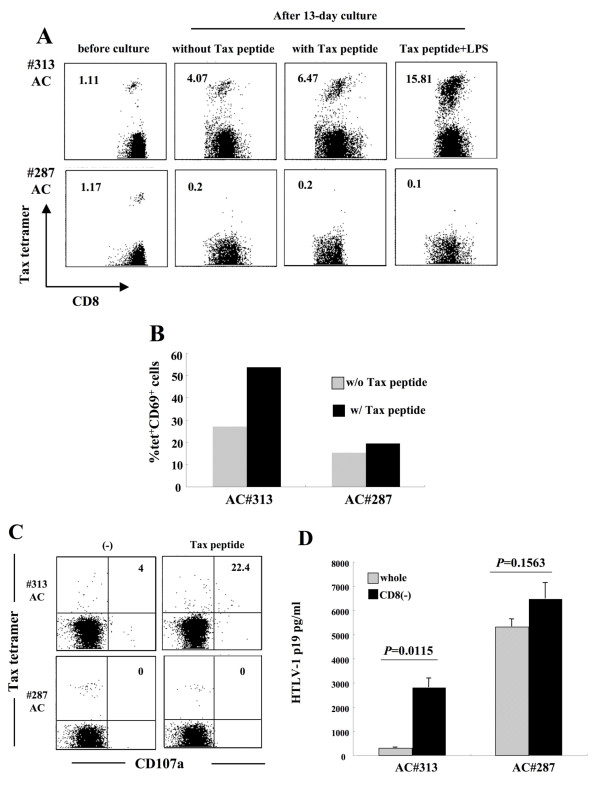
**Dysfunction of Tax-specific CD8^+ ^T-cells and inefficient CD8^+ ^cell-mediated HTLV-1 control in AC#287**. (A) For antigen-specific T-cell proliferation, PBMCs from #313 and #287 were cultured for 13 days with or without Tax peptide in the presence or absence of 0.1 μg/ml LPS. The number indicates the percentage of tetramer^+ ^cells in CD8^+ ^T-cells. (B, C) PBMCs were stimulated with or without 10 μM Tax peptide for 6 hrs. The expression of CD69 (B) and CD107a (C) in Tax-specific CD8^+ ^T-cells was analyzed by flow cytometry. (B) Bar indicates the percentage of CD69^+ ^cells in Tax-specific CD8^+ ^T-cells. (C) The number represents the percentage of CD107a^+ ^cells in Tax-specific CD8^+ ^T-cells. (D) Whole PBMCs and CD8-depleted fractions in ACs (#287 and #313) were cultured for 7 days and HTLV-1 p19 in the supernatants were measured by HTLV-1 p19 ELISA. *P *value was determined by the unpaired *t *test.

To examine whether Tax-specific CD8^+ ^T-cell responses were influenced by activation of antigen-presenting cells (APCs), PBMC from #313 (responder) and #287 (low responder) were stimulated with Tax peptide in the presence or absence of LPS, a potent activator of APCs such as dendritic cells (DCs) and monocytes/macrophages. In #313, the frequency of Tax-specific CD8^+ ^T-cells increased from 1.11% to 6.47% or 4.07% at day 13, after stimulation with or without Tax peptide, respectively. The frequency of Tax-specific CD8^+ ^T-cells in #313 further increased in the presence of Tax peptide and LPS (15.81%). In contrast to #313, the frequency of Tax-specific CD8^+ ^T-cells in #287 decreased from 1.17% to 0.2% after stimulation with Tax peptide, and was not recovered by LPS stimulation (Figure 4A). In addition, HTLV-1-infected cells have been reported to express C-C chemokine receptor type 4 (CCR4) and have FoxP3^+ ^Treg-like function[18,40]. However, the proliferative ability of Tax-specific CD8^+ ^T-cells in #287 was not restored even in the absence of CCR4^+ ^infected cells (data not shown).

To further examine the function of Tax-specific CD8^+ ^T-cells in #313 and #287, we observed the expression of CD69, an early activation marker transiently expressed on T lymphocytes that precedes cytokine secretion after antigenic stimulation, and CD107a, a marker of degranulation associated with cytotoxic activity in an antigen-specific manner[41]. CD69 was up-regulated on Tax-specific CD8^+ ^T-cells in #313 when stimulated with Tax peptide, but not in #287, which was in agreement with their abilities to produce IFN-γ (Figure 4B). In #313, 22.4% of Tax-specific CD8^+ ^T-cells mobilized CD107a to the surface during a 6-hr culture with Tax peptide stimulation, while CD107a surface expression was detected on 4% of Tax-specific CD8^+ ^T-cells in the culture without stimulation (Figure 4C). However, no CD107a mobilization was detected on the surface of Tax-specific CD8^+ ^T-cells in #287 with or without Tax peptide stimulation (Figure 4C). These results indicate that HTLV-1-specific CD8^+ ^T-cells in AC #287 did not properly activate upon antigen stimulation, and therefore failed to control HTLV-1-infected cells.

The Tax/HLA tetramers used in this study allow us to evaluate the functions of CD8^+ ^T-cells only against an immunodominant epitope, Tax. We therefore compared HTLV-1 Gag p19 in the culture between whole and CD8^+ ^cell-depleted PBMCs to examine the role of total HTLV-1-specific CD8^+ ^T-cells including the dominant Tax-specific CD8^+ ^T-cells, in suppression of HTLV-1 production from infected cells (Figure 4D). As expected, depletion of CD8^+ ^cells from PBMCs in #313 led to significantly higher HTLV-1 production compared to whole PBMCs (*P *= 0.0115). In contrast, HTLV-1 p19 production increased only a little in the culture of CD8^+ ^cell-depleted PBMCs in #287 (*P *= 0.1563), indicating that HTLV-1-specific CD8^+ ^T-cells other than the dominant Tax-specific CD8^+ ^T-cells might have a reduced ability to control the infected cells in this donor. It is of note that HTLV-1-infected cells from both two donors carried intact HTLV-1 proviral genomic DNA because HTLV-1 p19 could be detected after 7 day-culture.

### Phenotypic analysis of functional and dysfunctional Tax-specific CD8^+ ^T-cells

**Figure 5:**
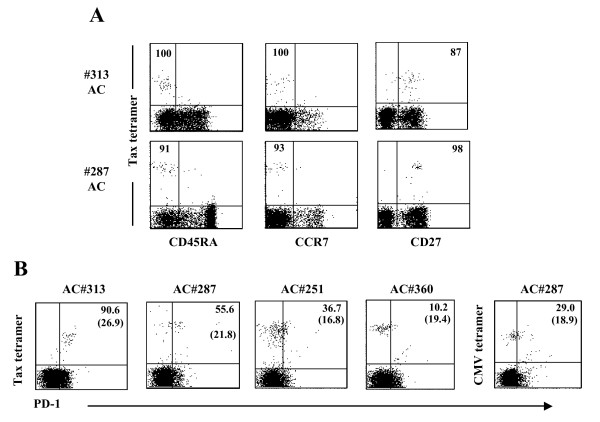
**Phenotypic analysis of functional and dysfunctional Tax-specific CD8^+ ^T-cells**. (A) Differentiation memory phenotype, based on the expression of CD45RA, CCR7, and CD27 and (B) PD-1 expression of Tax-specific CD8^+ ^T-cells from ACs were examined by flow cytometry. The number represents the percentage of indicated marker-positive or -negative cells in tetramer^+ ^CD8^+ ^T-cells. The number given in parenthesis shows MFI of PD-1 expression on the PD-1^+ ^tetramer^+ ^cells.

We next characterized the differentiation status of memory T-cells in Tax-specific CD8^+ ^T-cells. Human CD8 T-cells may be classified as naïve T-cells (CD45RA^+^CCR7^+^CD27^+^), T_CM _(CD45RA^-^CCR7^+^CD27^+^), T_EM _(CD45RA^-^CCR7^-^CD27^+^), and T_Diff _(CD45RA^+^CCR7^-^CD27^-^) cells[42-44]. As shown in Figure 5A, almost all Tax-specific CD8^+ ^T-cells in both #313 and #287 were skewed to CD45RA^-^CCR7^-^CD27^+ ^T_EM _cells, and there was no essential difference between two donors.

A previous report has shown that PD-1 was highly up-regulated on Tax-specific CD8^+ ^T-cells in ATL patients and ACs[32]. We therefore examined PD-1 expression on Tax-specific CD8^+ ^T-cells in several AC samples, including #287. The frequency of PD-1^+ ^Tax-specific CD8^+ ^T-cells was very high in #309 (85.3%) and #313 (96%) (Figure 5B and Table 2) while those Tax-specific CD8^+ ^T-cells retained the proliferative and the cytokine-producing abilities (Figure 3A and Table 2). In #287, the frequency of PD-1-expressing Tax-specific CD8^+ ^T-cells (55.6%) was lower than #309 and #313, but higher than that of PD-1^+ ^CMVpp65-specific CD8^+ ^T-cells in the same donor (Figure 5B). The levels of PD-1 expression showed a similar tendency to the frequency of PD-1^+ ^T-cells. In addition, the blockade of PD-1/PD-ligand 1 (PD-L1) pathway did not restore the proliferative capacity of Tax-specific CD8^+ ^T-cells in #287 (data not shown).

### Conserved functions of CMV-specific CD8^+ ^T-cells in #287

**Figure 6:**
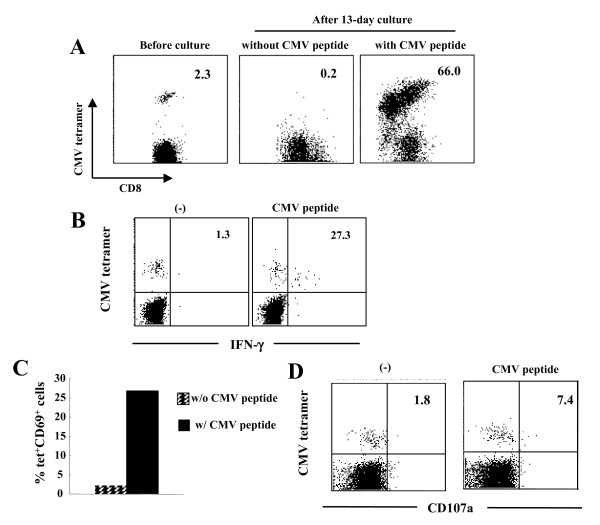
**Conserved functions of CMV-specific CD8^+ ^T-cells in AC#287**. (A) For antigen-specific T-cell proliferation, PBMCs from #287 were cultured for 13 days with or without 100 nM CMV peptide. The number indicates the percentage of CMV tetramer^+ ^cells in CD8^+ ^T-cells. (B-D) PBMCs were stimulated with or without 10 μM CMV peptide for 6 hrs. IFN-γ production (B), CD69 (C) and CD107a (D) expression of CMVpp65-specific CD8^+ ^T-cells in #287 was analyzed by flow cytometry. (B, D) The number represents the percentage of the indicated marker-positive cells in CMVpp65-specific CD8^+ ^T-cells. (C) Bar indicates the percentage of CD69^+ ^cells in CMV-specific CD8^+ ^T-cells.

We next examined whether the impairment of proliferative capacity and effector functions observed in #287 CD8^+ ^T-cells were specific for HTLV-1 antigens or the result of general immune suppression. PBMC from #287 contained CMVpp65-specific CD8^+ ^T-cells (2.3% of CD8^+ ^T-cells), as detected by tetramer staining. The frequency of CMVpp65-specific CD8^+ ^T-cells increased from 2.3% to 66.0% following in vitro CMVpp65 peptide stimulation, but not without the peptide stimulation (Figure 6A). Antigen-specific IFN-γ and CD69 expression were clearly detected in CMVpp65-specific CD8^+ ^T-cells in #287 (Figures 6B and 6C). Furthermore, CMVpp65-specific CD8^+ ^T-cells mobilized CD107a to the surface in response to CMVpp65 peptide (Figure 6D). These results demonstrate that in #287, CMVpp65-specific CD8^+ ^T-cells, but not Tax-specific CD8^+ ^T-cells, have proliferative potential and effector functions, such as cytotoxic activity and IFN-γ release, suggesting that the impaired CD8^+ ^T-cell function in #287 was specific for HTLV-1.

### Dysfunction of Tax-specific but not CMVpp65-specific CD8^+ ^T-cells also in sATL patients

**Figure 7:**
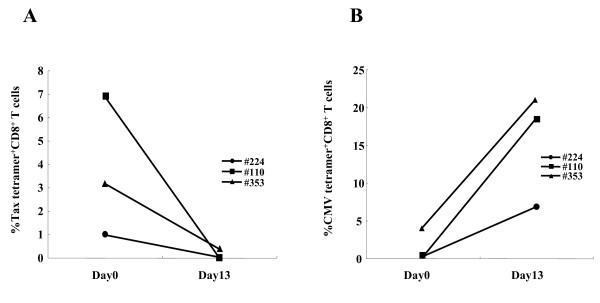
**Impaired proliferation of Tax-specific but not CMVpp65-specific CD8^+ ^T-cells in sATL patients**. For antigen-specific T-cell proliferation, PBMCs from sATL (#110; square, #353; triangle) and cATL (#224; circle) patients were cultured for 13 days with 100 nM Tax (A) or CMV (B) peptide. Each dot indicates the percentage of tetramer^+ ^cells in CD8^+ ^T-cells at day 0 and day 13 after culture. Clinical information on ATL patients used here is as follows; sATL#110: age; 40 s, gender; F, WBC#; 11,000/μL {lymphocyte (lym); 39%, abnormal lymphocytes (ably); 4%}, cATL#224: age; 50 s, gender; F, WBC#; 7900/μL (lym; 30%, ably; 33%), sATL#353: age; 60 s, gender; M, WBC#; 4620/μL (lym; 39%, ably; 5%).

Finally, we extended the study to see whether patients with early stage ATL might exhibit similar dysfunction selective for HTLV-1-specific CD8^+ ^T-cells. We found two smoldering ATL (sATL) patients (#110 and #353) possessing 6.89% and 3.15% of tetramer-binding Tax-specific CD8^+ ^T-cells, respectively. The sATL patient #353 carried 5% of abnormal lymphocytes (ably) with a normal range of lymphocyte number, whose status is very close to the borderline with ACs. Patient #110 carried 4% of abnormal lymphocytes with mild lymphocytosis. Tax-specific CD8^+ ^T-cells of two sATL patients (#110 and #353) did not proliferate in response to Tax peptides as similarly observed in a cATL patient (#224) (Figure 7A) and most other cATL patients (Figure 2A and Additional file 1). In contrast, CMVpp65-specific CD8^+ ^T-cells in both sATL patients vigorously proliferated when stimulated with CMVpp65 peptides. CMVpp65-specific CD8^+ ^T-cells in a cATL (#224) also proliferated, but to a lesser degree, which might reflect general immune suppression in this patient (Figure 7).

## Discussion

In this study, we detected Tax-specific CD8^+ ^T-cells in 87%, but not the rest of ACs tested, by using tetramers containing Tax major epitope-peptides presented by HLA-A*0201, A*1101, and A*2402. Tax-specific CD8^+ ^T-cells were also detected in 38% of cATL patients, but at reduced frequencies and with severely impaired functions. Further analysis of Tax-specific CD8^+ ^T-cells in 14 ACs indicated that they were functional in most of ACs tested except one (#287), whose Tax-specific CD8^+ ^T-cells poorly responded to specific peptides. However, CMVpp65-specific CD8^+ ^T-cells of this individual were fully functional. Similar T-cell dysfunction selective for HTLV-1, but not CMV, was also observed in sATL patients, one of which (#353) had no clinical symptoms but 5% abnormal lymphocytes. General immune suppression might partly account for the scarcity and/or the dysfunction of Tax-specific CD8^+ ^T-cells in ATL patients, but not those in the AC or the sATL patients as they were selective for HTLV-1. These findings suggest that HTLV-1-specific immune suppression is undergoing in a minor group of ACs and an early stage of ATL.

The presence of tetramer-binding Tax-specific CD8^+ ^T-cells in cATL patients, although at low frequencies, implies that they have encountered antigen during the chronic phase of ATL disease, suggesting that Tax may be expressed *in vivo*. This may be supported by a previous report showing that virus-specific CD8^+ ^T-cells fails to acquire memory T-cell property of long-term antigen-independent persistence during chronic lymphocytic choriomeningitis virus (LCMV) infection[45]. However, there is no direct evidence that infected cells produce Tax in infected individuals. HTLV-1-specific T-cell responses in cATL patients are largely different from HAM/TSP patients. In HAM/TSP patients, Tax-specific CD8^+ ^T-cells proliferated vigorously and a large population of them produced IFN-γ. In contrast, the function of Tax-specific CD8^+ ^T-cells in cATL patients was profoundly suppressed, similarly to tumor infiltrating lymphocytes (TIL)[46]. In cATL patients, Tax-specific CD8^+ ^T-cells that were detected before culture decreased in number to undetectable or very low levels after 6 days, regardless of peptide stimulation (data not shown). This is not likely to be due to TCR down-regulation, because TCRs on Tax-specific CD8^+ ^T-cells in HAM/TSP patients are down-regulated on days 1 to 4 and reappeared by day 6 in vitro[34]. Moreover, we could not observe any tetramer^+ ^CD8^+ ^T-cells even in the 13-day culture (data not shown), suggesting these cells might have died during the culture.

Severe dysfunction of Tax-specific CD8^+ ^T-cells was observed not only in cATL patients, but also in an AC #287. Fresh PBMCs of #287 contained 1.17% tetramer^+ ^cells in the CD8^+ ^T-cell fraction. However, none of these tetramer-positive T-cells proliferated in culture, with or without Tax peptide stimulation (Figure 3B). Although a few populations of them (11.1%) produced a small amount of IFN-γ, they lacked degranulation activity for cytotoxicity or expression of CD69, an early activation marker, upon specific stimulation (Figures 3 and 4). Importantly, CMVpp65-specific CD8^+ ^T-cells in the same donor were clearly activated, and exhibited these characteristics upon stimulation with pp65 peptides (Figure 6). These observations indicated that the impaired Tax-specific CD8^+ ^T-cells function in #287 was not attributable to general immune suppression, but to an HTLV-1-specific phenomenon. In addition, CD8-depletion study indicated that not only the dominant Tax-specific CD8^+ ^T-cell function but also other HTLV-1-specific CD8^+ ^T cell responses might be reduced in #287 (Figure 4D). Since CMV-specific CD8^+ ^T-cells responded well to the specific peptides, antigen-presenting cells in culture were not likely to be responsible for the selective suppression of Tax-specific CD8^+ ^T-cells. In addition, it has been shown that HTLV-1-infected cells generally express CCR4 and have Treg-like function[18,40]. However, depletion of CCR4^+ ^cells did not restore the proliferative ability of Tax-specific CD8^+ ^T-cells (data not shown), indicating that suppression of the infected cells were not likely to be the major reason for the impaired Tax-specific CD8^+ ^T-cell function in our culture system. These observations suggest that in #287, Tax-specific CD8^+ ^T-cells themselves might lose their functions.

Many chronic viral infections affect the phenotype, function, and maintenance of memory T-cells[24,42,47,48]. T_EM _cells predominate in infections in which relatively high levels of antigen persist and continuous antigen stimulation are required for maintenance of T_EM _cells. As described in HAM/TSP patients[34], Tax-specific CD8^+ ^T-cells in both ACs (#287 and #313) were primarily enriched in T_EM _memory pool in spite of the functionality of Tax-specific CD8^+ ^T-cells (Figure 5A), which may support continuous or periodical expression of viral antigen in vivo during an asymptomatic stage.

PD-1 is known to play a major role in regulating T-cell exhaustion during chronic infection. In this study, we could not obtain any data supporting the involvement of PD-1 in the dysfunction of Tax-specific CD8^+ ^T-cells. However, we observed that Tax-specific CD8^+ ^T-cells in some ACs showed IFN-γ production, but not proliferative capacity (Table 2). This partially lacked function of Tax-specific CD8^+ ^T-cells is similar to the features of T-cell exhaustion. Whether Tax-specific CD8^+ ^T-cells are exhausted in HTLV-1 infection, and whether other molecules associated with T-cell exhaustion are involved in the impairment of Tax-specific CD8^+ ^T-cell responses are necessary to be clarified because some inhibitory molecules such as T-cell immunoglobulin and mucin domain-containing protein-3 (TIM-3), lymphocyte activated gene-3 (LAG-3), and transcription factors including BLIMP-1 are also found to be associated with T-cell exhaustion [49].

The incidence of Tax-specific CD8^+ ^T-cell detection was high (87.0%) in ACs. Given the fact that the incidence of Tax-specific CD8^+ ^T-cells in HAM/TSP patients was 100%, a small fraction of ACs lacking detectable tetramer-binding cells might lack Tax-specific T-cell responses. Our previous study investigating GST-Tax protein-based T-cell responses supports this notion [20]. In the present study, even in ACs possessing Tax-specific CD8^+ ^T-cells, at least one individual exhibited T-cell dysfunction selectively for HTLV-1. The incidence of tetramer-positive cells was reduced in ATL patients (38.1%), and the function of these cells was impaired in all the ATL patients even with detectable tetramer-binding Tax-specific CD8^+ ^T-cells. Our findings suggest that HTLV-1-specific T-cell responses are selectively impaired in a small percentage of HTLV-1-infected individuals in the asymptomatic stages, and the proportion of individuals with such characteristics increase as the stages proceed towards ATL. Strategies to reactivate HTLV-1-specific T-cells at early stages might contribute to a reduction in the immunological risk of ATL.

## Conclusions

Tax-specific CD8^+ ^T-cells were scarce and dysfunctional in a limited AC population and ATL patients, and the dysfunction of CD8^+ ^T-cells was selective for HTLV-1 in early stages. These results implied the presence of some HTLV-1-specific T-cell suppressive mechanisms even in asymptomatic stages, which are not a result of general immune suppression in ATL but could be underlying conditions toward disease progression.

## Methods

### Samples

Blood samples from 64 HTLV-1-seropositive individuals were used in this study: 23 asymptomatic carriers (ACs), 18 HAM/TSP patients, 2 smoldering type ATL (sATL) patients, and 21 chronic type ATL (cATL) patients. All blood samples were obtained following written informed consent, and this study was reviewed and approved by the Institutional Review Board of the Tokyo Medical and Dental University.

### Peptides

Peptides used in this study were HLA-A2-restricted CTL epitopes (Tax11-19, LLFGYPVYV)[12] (Hokudo Co., Hokkaido, Japan) and (CMV495-503, NLVPMVATV)[50] (Sigma Aldrich St. Louis, MO), HLA-A11-restricted CTL epitope (Tax88-96, KVLTPPITH)[36] (Hokudo Co) and HLA-A24-restricted CTLs epitopes (Tax301-309, SFHSLHLF)[35] (Hokudo Co) and (CMV341-349, QYDPVAALF)[51] (Sigma Aldrich).

### Cell Surface staining

To select samples carrying HLA-A2, -A11, or -A24, whole blood was screened with antibodies for HLA-A2, -A11, and -A24 subtypes (One Lambda, Inc., Los Angeles, CA). FITC-conjugated goat anti-mouse Ig (G+M) (Beckman Coulter Inc., Webster, TX) was used as a secondary antibody. For cell surface staining, whole blood samples were stained with the following fluorochrome-conjugated mouse anti-human mAbs; CD3-FITC, CD8-PE/Cy5, CD8-PerCP/Cy5.5 (RPA-T8, BioLegend), CD27-FITC (O323, BioLegend) CD45RA-FITC (HI 100, BD Biosciences), CD45RA-APC (HI 100, BioLegend), CD69-FITC (FN 50, BioLegend), PD-1-FITC (EH12.2H7, BioLegend), CCR7 (TG8/CCR7, Biolegend).

### Tetramer staining

PE-conjugated HLA-A*0201/Tax11-19, HLA-A*1101/Tax88-96, HLA-A*2402/Tax301-309, HLA-A*0201/CMVpp65, HLA-A*2402/CMVpp65 tetramers were purchased from MBL (Nagoya, Japan). Whole blood samples or peripheral blood mononuclear cells (PBMCs) were stained with PE-conjugated Tax/HLA tetramer in conjunction with FITC-conjugated anti-CD3 (UCHT1, BioLegend San Diego, CA), and PE-Cy5-conjugated anti-CD8 monoclonal antibodies (mAbs) (HIT8a, BD Biosciences San Jose, CA). Whole blood samples were lysed and fixed in BD FACS lysing solution (BD Biosciences) before washing the cells. Samples were analyzed on a FACSCalibur (Becton Dickinson, San Jose, CA) and data analyses were performed using CellQuest software (Becton Dickinson).

### Tetramer-based IFN-γ flow cytometry

Tetramer-based intracellular IFN-γ flow cytometry was performed as described previously[17], with slight modifications. In brief, PBMCs (2.0 × 10^5 ^cells) were incubated with HLA tetramer-PE and anti-CD8-PE/Cy5, washed, and stimulated with 10 μM antigenic peptide for 6 hrs at 37°C in the presence of brefeldin A (BFA, 10 μg/ml; Sigma Aldrich). The cells were stained with a tetramer, permeabilized, and stained with anti-human IFN-γ-FITC (4S.B3, BD Biosciences).

### T-cell proliferation

PBMCs (2.0-5.0 × 10^5 ^cells/well) labeled with carboxyfluorescein succinimidyl ester (CFSE; Sigma Aldrich) were cultured for 6 days with or without 100 nM antigenic peptide and then stained with Tax/HLA tetramer-PE and anti-CD8-PE/Cy5. In some experiments, PBMCs (2.0 × 10^5 ^cells) were cultured for 13 days with 100 nM antigenic peptide and 10 U/ml recombinant human IL-2 (IL-2; Shionogi, Osaka, Japan) in the presence or absence of 0.1 μg/ml Lipopolysaccharide (LPS; Sigma Aldrich). The cells were then stained with HLA tetramer-PE, anti-CD8-PE/Cy5 and anti-CD3-FITC, and analyzed by flow cytometry.

### Quantification of HTLV-1 proviral load

The HTLV-1 proviral load was measured using LightCycler DNA Master SYBR Green 1 (Roche, Mannheim, Germany) with a LightCycler (Roche). Genomic DNA was extracted from PBMCs (2 × 10^6 ^cells) using DNeasy Blood & Tissue kits (QIAGEN, Courtaboeuf, France). The primer sets used in this study were as follows: pX2 (5'-CGGATACCCAGTCTACGTGTTTGGAGACTGT-3') and pX3 (5'-GAGCCGATAACGCGTCCATCGATGGGGTCC-3') for HTLV-1 pX, and B-globin (5'-ACACAACTGTGTTCACTAGC-3') and aB-globin (5'-CAACTTCATCCACGTTCACC-3') for β-globin. The proviral load was calculated as: [(copy number of pX)/(copy number of β-globin/2)] × 1000. HTLV-1 proviral loads in some of the PBMC samples were measured by the Group of Joint Study on Predisponsing Factors of ATL Development (JSPFAD, Japan) as described previously [20].

### CD107a mobilization assay

PBMCs were stained with Tax/HLA tetramers-PE and anti-CD8-PE/Cy5, washed, and stimulated with 10 μM antigenic peptide for 6 hrs at 37°C in the presence of mouse anti-human CD107a-PerCP/Cy5.5 (H4A3, Biolegend) or mouse IgG_1_-PerCP/Cy5.5 (MOPC-21, Biolegend). BFA (10 μg/ml) was added 1 hr after incubation was started. The cells were then collected and stained with an HLA tetramer.

### Depletion of CD8^+ ^cells and Detection of HTLV-1 p19

CD8^+ ^cells were depleted from PBMCs by negative selection using 10-fold numbers of Dynabeads M-450 CD8 (Invitrogen, Carlsbad, CA), according to the manufacturer's instructions. The PBMCs were adjust to 1 × 10^6 ^cells/ml before depletion, and the resulting CD8^+ ^cell-depleted fractions were resuspended in medium with the same initial volume, irrespective of the remaining cell number. PBMCs (1 × 10^6 ^cells/ml) and CD8^+ ^cell-depleted PBMCs were cultured for 7 days. HTLV-1 p19 in the supernatants of those PBMCs were measured by HTLV p19 antigen ELISA (RETRO tek, Buffalo, NY).

### Statistics

The Mann-Whitney U-test, the unpaired t test, and the Spearman rank correlation test were performed for statistical significance by using the Graphpad Prism software (Graphpad Software). In all cases, two-tailed *P *values less than 0.05 were considered significant.

## Competing interests

The authors declare that they have no competing interests.

## Authors' contributions

AT carried out immunological and virological analyses, and drafted the manuscript. AH conceived of the study, participated in its design and coordination, and drafted the manuscript. AU, YM, YY, MM, IC, NU, and JO provided clinical samples. YS, YT, AS, and NZ carried out a part of the experiments. TW provided the data on proviral load of some HTLV-1-infected individuals. TM helped to draft the manuscript. MK participated in study design and helped to draft the manuscript. All authors read and approved the final manuscript.

## Supplementary Material

Additional file 1 **Tax-specific CD8^+ ^T-cells in cATL patients could not proliferate against Tax-peptide stimulation**. (A) CFSE-labeled PBMCs were cultured with or without 100 nM Tax-peptide for 6 days. The number indicates the percentage of tetramer^+ ^cells in CD8^+ ^T cells (Day 0) or the percentage of dividing (CFSE ^low^) cells in Tax-specific CD8^+ ^T-cells (Day 6). In a cATL sample #54, CFSE-labeled PBMCs were cultured in the presence of mouse IgG for other experiment. (B) PBMCs (#224) and CCR4-depleted PBMCs (#280) were cultured for 13 days in the presence of 100 nM Tax-peptide. The number indicates the percentage of tetramer^+ ^cells in CD8^+ ^T-cells. Click here for file

## References

[B1] Hinuma Y Nagata K Hanaoka M Nakai M Matsumoto T Kinoshita KI Shirakawa S Miyoshi I Adult T-cell leukemia: antigen in an ATL cell line and detection of antibodies to the antigen in human sera Proc Natl Acad Sci USA 1981 78 6476 6480 10.1073/pnas.78.10.6476 PMC349062 7031654

[B2] Poiesz BJ Ruscetti FW Gazdar AF Bunn PA Minna JD Gallo RC Detection and isolation of type C retrovirus particles from fresh and cultured lymphocytes of a patient with cutaneous T-cell lymphoma Proc Natl Acad Sci USA 1980 77 7415 7419 10.1073/pnas.77.12.7415 PMC350514 6261256

[B3] de The G Bomford R An HTLV-I vaccine: why, how, for whom? AIDS Res Hum Retroviruses 1993 9 381 386 10.1089/aid.1993.9.381 8318266

[B4] Arisawa K Soda M Endo S Kurokawa K Katamine S Shimokawa I Koba T Takahashi T Saito H Doi H Shirahama S Evaluation of adult T-cell leukemia/lymphoma incidence and its impact on non-Hodgkin lymphoma incidence in southwestern Japan Int J Cancer 2000 85 319 324 10.1002/(sici)1097-0215(20000201)85:3<319::aid-ijc4>3.0.co;2-b 10652420

[B5] Gessain A Barin F Vernant JC Gout O Maurs L Calender A de The G Antibodies to human T-lymphotropic virus type-I in patients with tropical spastic paraparesis Lancet 1985 2 407 410 10.1016/s0140-6736(85)92734-5 2863442

[B6] Osame M Izumo S Igata A Matsumoto M Matsumoto T Sonoda S Tara M Shibata Y Blood transfusion and HTLV-I associated myelopathy Lancet 1986 2 104 105 10.1016/s0140-6736(86)91636-3 2873363

[B7] Tajima K The 4th nation-wide study of adult T-cell leukemia/lymphoma (ATL) in Japan: estimates of risk of ATL and its geographical and clinical features. The T- and B-cell Malignancy Study Group Int J Cancer 1990 45 237 243 10.1002/ijc.2910450206 2303290

[B8] Uchiyama T Yodoi J Sagawa K Takatsuki K Uchino H Adult T-cell leukemia: clinical and hematologic features of 16 cases Blood 1977 50 481 492 301762

[B9] Bangham CR HTLV-1 infection: role of CTL efficiency Blood 2008 112 2176 2177 10.1182/blood-2008-06-163071 PMC2577558 18779398

[B10] Jacobson S Shida H McFarlin DE Fauci AS Koenig S Circulating CD8+ cytotoxic T lymphocytes specific for HTLV-I pX in patients with HTLV-I associated neurological disease Nature 1990 348 245 248 10.1038/348245a0 2146511

[B11] Bieganowska K Hollsberg P Buckle GJ Lim DG Greten TF Schneck J Altman JD Jacobson S Ledis SL Hanchard B Chin J Morgan O Roth PA Hafler DA Direct analysis of viral-specific CD8+ T cells with soluble HLA-A2/Tax11-19 tetramer complexes in patients with human T cell lymphotropic virus-associated myelopathy J Immunol 1999 162 1765 1771 9973440

[B12] Kannagi M Shida H Igarashi H Kuruma K Murai H Aono Y Maruyama I Osame M Hattori T Inoko H Target epitope in the Tax protein of human T-cell leukemia virus type I recognized by class I major histocompatibility complex-restricted cytotoxic T cells J Virol 1992 66 2928 2933 10.1128/jvi.66.5.2928-2933.1992 PMC241051 1373197

[B13] Parker CE Daenke S Nightingale S Bangham CR Activated, HTLV-1-specific cytotoxic T-lymphocytes are found in healthy seropositives as well as in patients with tropical spastic paraparesis Virology 1992 188 628 636 10.1016/0042-6822(92)90517-s 1374983

[B14] Parker CE Nightingale S Taylor GP Weber J Bangham CR Circulating anti-Tax cytotoxic T lymphocytes from human T-cell leukemia virus type I-infected people, with and without tropical spastic paraparesis, recognize multiple epitopes simultaneously J Virol 1994 68 2860 2868 10.1128/jvi.68.5.2860-2868.1994 PMC236774 7512153

[B15] Uchiyama T Human T cell leukemia virus type I (HTLV-I) and human diseases Annu Rev Immunol 1997 15 15 37 10.1146/annurev.immunol.15.1.15 9143680

[B16] Arnulf B Thorel M Poirot Y Tamouza R Boulanger E Jaccard A Oksenhendler E Hermine O Pique C Loss of the ex vivo but not the reinducible CD8+ T-cell response to Tax in human T-cell leukemia virus type 1-infected patients with adult T-cell leukemia/lymphoma Leukemia 2004 18 126 132 10.1038/sj.leu.2403176 14574331

[B17] Kozako T Arima N Toji S Masamoto I Akimoto M Hamada H Che XF Fujiwara H Matsushita K Tokunaga M Haraguchi K Uozumi K Suzuki S Takezaki T Sonoda S Reduced frequency, diversity, and function of human T cell leukemia virus type 1-specific CD8+ T cell in adult T cell leukemia patients J Immunol 2006 177 5718 5726 10.4049/jimmunol.177.8.5718 17015761

[B18] Chen S Ishii N Ine S Ikeda S Fujimura T Ndhlovu LC Soroosh P Tada K Harigae H Kameoka J Kasai N Sasaki T Sugamura K Regulatory T cell-like activity of Foxp3+ adult T cell leukemia cells Int Immunol 2006 18 269 277 10.1093/intimm/dxh366 16361311

[B19] Hishizawa M Imada K Kitawaki T Ueda M Kadowaki N Uchiyama T Depletion and impaired interferon-alpha-producing capacity of blood plasmacytoid dendritic cells in human T-cell leukaemia virus type I-infected individuals Br J Haematol 2004 125 568 575 10.1111/j.1365-2141.2004.04956.x 15147371

[B20] Shimizu Y Takamori A Utsunomiya A Kurimura M Yamano Y Hishizawa M Hasegawa A Kondo F Kurihara K Harashima N Watanabe T Okamura J Masuda T Kannagi M Impaired Tax-specific T-cell responses with insufficient control of HTLV-1 in a subgroup of individuals at asymptomatic and smoldering stages Cancer Sci 2009 100 481 489 10.1111/j.1349-7006.2008.01054.x PMC11158518 19154412

[B21] Gruener NH Lechner F Jung MC Diepolder H Gerlach T Lauer G Walker B Sullivan J Phillips R Pape GR Klenerman P Sustained dysfunction of antiviral CD8+ T lymphocytes after infection with hepatitis C virus J Virol 2001 75 5550 5558 10.1128/JVI.75.12.5550-5558.2001 PMC114267 11356962

[B22] Klenerman P Hill A T cells and viral persistence: lessons from diverse infections Nat Immunol 2005 6 873 879 10.1038/ni1241 16116467

[B23] Kostense S Vandenberghe K Joling J Van Baarle D Nanlohy N Manting E Miedema F Persistent numbers of tetramer+ CD8(+) T cells, but loss of interferon-gamma+ HIV-specific T cells during progression to AIDS Blood 2002 99 2505 2511 10.1182/blood.v99.7.2505 11895786

[B24] Shankar P Russo M Harnisch B Patterson M Skolnik P Lieberman J Impaired function of circulating HIV-specific CD8(+) T cells in chronic human immunodeficiency virus infection Blood 2000 96 3094 3101 11049989

[B25] Wherry EJ Blattman JN Murali-Krishna K van der Most R Ahmed R Viral persistence alters CD8 T-cell immunodominance and tissue distribution and results in distinct stages of functional impairment J Virol 2003 77 4911 4927 10.1128/JVI.77.8.4911-4927.2003 PMC152117 12663797

[B26] Zajac AJ Blattman JN Murali-Krishna K Sourdive DJ Suresh M Altman JD Ahmed R Viral immune evasion due to persistence of activated T cells without effector function J Exp Med 1998 188 2205 2213 10.1084/jem.188.12.2205 PMC2212420 9858507

[B27] Day CL Kaufmann DE Kiepiela P Brown JA Moodley ES Reddy S Mackey EW Miller JD Leslie AJ DePierres C Mncube Z Duraiswamy J Zhu B Eichbaum Q Altfeld M Wherry EJ Coovadia HM Goulder PJ Klenerman P Ahmed R Freeman GJ Walker BD PD-1 expression on HIV-specific T cells is associated with T-cell exhaustion and disease progression Nature 2006 443 350 354 10.1038/nature05115 16921384

[B28] Radziewicz H Ibegbu CC Fernandez ML Workowski KA Obideen K Wehbi M Hanson HL Steinberg JP Masopust D Wherry EJ Altman JD Rouse BT Freeman GJ Ahmed R Grakoui A Liver-infiltrating lymphocytes in chronic human hepatitis C virus infection display an exhausted phenotype with high levels of PD-1 and low levels of CD127 expression J Virol 2007 81 2545 2553 10.1128/JVI.02021-06 PMC1865979 17182670

[B29] Zhang JY Zhang Z Wang X Fu JL Yao J Jiao Y Chen L Zhang H Wei J Jin L Shi M Gao GF Wu H Wang FS PD-1 up-regulation is correlated with HIV-specific memory CD8+ T-cell exhaustion in typical progressors but not in long-term nonprogressors Blood 2007 109 4671 4678 10.1182/blood-2006-09-044826 17272504

[B30] Barber DL Wherry EJ Masopust D Zhu B Allison JP Sharpe AH Freeman GJ Ahmed R Restoring function in exhausted CD8 T cells during chronic viral infection Nature 2006 439 682 687 10.1038/nature04444 16382236

[B31] Freeman GJ Long AJ Iwai Y Bourque K Chernova T Nishimura H Fitz LJ Malenkovich N Okazaki T Byrne MC Horton HF Fouser L Carter L Ling V Bowman MR Carreno BM Collins M Wood CR Honjo T Engagement of the PD-1 immunoinhibitory receptor by a novel B7 family member leads to negative regulation of lymphocyte activation J Exp Med 2000 192 1027 1034 10.1084/jem.192.7.1027 PMC2193311 11015443

[B32] Kozako T Yoshimitsu M Fujiwara H Masamoto I Horai S White Y Akimoto M Suzuki S Matsushita K Uozumi K Tei C Arima N PD-1/PD-L1 expression in human T-cell leukemia virus type 1 carriers and adult T-cell leukemia/lymphoma patients Leukemia 2009 23 375 382 10.1038/leu.2008.272 18830259

[B33] van Lier RA ten Berge IJ Gamadia LE Human CD8(+) T-cell differentiation in response to viruses Nat Rev Immunol 2003 3 931 939 10.1038/nri1254 14647475

[B34] Johnson-Nauroth JM Graber J Yao K Jacobson S Calabresi PA Memory lineage relationships in HTLV-1-specific CD8+ cytotoxic T cells J Neuroimmunol 2006 176 115 124 10.1016/j.jneuroim.2006.03.013 PMC4988392 16740321

[B35] Harashima N Kurihara K Utsunomiya A Tanosaki R Hanabuchi S Masuda M Ohashi T Fukui F Hasegawa A Masuda T Takaue Y Okamura J Kannagi M Graft-versus-Tax response in adult T-cell leukemia patients after hematopoietic stem cell transplantation Cancer Res 2004 64 391 399 10.1158/0008-5472.can-03-1452 14729650

[B36] Harashima N Tanosaki R Shimizu Y Kurihara K Masuda T Okamura J Kannagi M Identification of two new HLA-A*1101-restricted tax epitopes recognized by cytotoxic T lymphocytes in an adult T-cell leukemia patient after hematopoietic stem cell transplantation J Virol 2005 79 10088 10092 10.1128/JVI.79.15.10088-10092.2005 PMC1181560 16014972

[B37] Elovaara I Koenig S Brewah AY Woods RM Lehky T Jacobson S High human T cell lymphotropic virus type 1 (HTLV-1)-specific precursor cytotoxic T lymphocyte frequencies in patients with HTLV-1-associated neurological disease J Exp Med 1993 177 1567 1573 10.1084/jem.177.6.1567 PMC2191033 8496677

[B38] Hanon E Hall S Taylor GP Saito M Davis R Tanaka Y Usuku K Osame M Weber JN Bangham CR Abundant tax protein expression in CD4+ T cells infected with human T-cell lymphotropic virus type I (HTLV-I) is prevented by cytotoxic T lymphocytes Blood 2000 95 1386 1392 10666215

[B39] Sakai JA Nagai M Brennan MB Mora CA Jacobson S In vitro spontaneous lymphoproliferation in patients with human T-cell lymphotropic virus type I-associated neurologic disease: predominant expansion of CD8+ T cells Blood 2001 98 1506 1511 10.1182/blood.v98.5.1506 11520801

[B40] Yoshie O Fujisawa R Nakayama T Harasawa H Tago H Izawa D Hieshima K Tatsumi Y Matsushima K Hasegawa H Kanamaru A Kamihira S Yamada Y Frequent expression of CCR4 in adult T-cell leukemia and human T-cell leukemia virus type 1-transformed T cells Blood 2002 99 1505 1511 10.1182/blood.v99.5.1505 11861261

[B41] Betts MR Brenchley JM Price DA De Rosa SC Douek DC Roederer M Koup RA Sensitive and viable identification of antigen-specific CD8+ T cells by a flow cytometric assay for degranulation J Immunol Methods 2003 281 65 78 10.1016/s0022-1759(03)00265-5 14580882

[B42] Appay V Dunbar PR Callan M Klenerman P Gillespie GM Papagno L Ogg GS King A Lechner F Spina CA Little S Havlir DV Richman DD Gruener N Pape G Waters A Easterbrook P Salio M Cerundolo V McMichael AJ Rowland-Jones SL Memory CD8+ T cells vary in differentiation phenotype in different persistent virus infections Nat Med 2002 8 379 385 10.1038/nm0402-379 11927944

[B43] Klebanoff CA Gattinoni L Restifo NP CD8+ T-cell memory in tumor immunology and immunotherapy Immunol Rev 2006 211 214 224 10.1111/j.0105-2896.2006.00391.x PMC1501075 16824130

[B44] Sallusto F Lenig D Forster R Lipp M Lanzavecchia A Two subsets of memory T lymphocytes with distinct homing potentials and effector functions Nature 1999 401 708 712 10.1038/44385 10537110

[B45] Wherry EJ Barber DL Kaech SM Blattman JN Ahmed R Antigen-independent memory CD8 T cells do not develop during chronic viral infection Proc Natl Acad Sci USA 2004 101 16004 16009 10.1073/pnas.0407192101 PMC524220 15505208

[B46] Radoja S Saio M Schaer D Koneru M Vukmanovic S Frey AB CD8(+) tumor-infiltrating T cells are deficient in perforin-mediated cytolytic activity due to defective microtubule-organizing center mobilization and lytic granule exocytosis J Immunol 2001 167 5042 5051 10.4049/jimmunol.167.9.5042 11673513

[B47] Champagne P Ogg GS King AS Knabenhans C Ellefsen K Nobile M Appay V Rizzardi GP Fleury S Lipp M Forster R Rowland-Jones S Sekaly RP McMichael AJ Pantaleo G Skewed maturation of memory HIV-specific CD8 T lymphocytes Nature 2001 410 106 111 10.1038/35065118 11242051

[B48] Penna A Pilli M Zerbini A Orlandini A Mezzadri S Sacchelli L Missale G Ferrari C Dysfunction and functional restoration of HCV-specific CD8 responses in chronic hepatitis C virus infection Hepatology 2007 45 588 601 10.1002/hep.21541 17326153

[B49] Yi JS Cox MA Zajac AJ T-cell exhaustion: characteristics, causes and conversion Immunology 2010 129 474 481 10.1111/j.1365-2567.2010.03255.x PMC2842494 20201977

[B50] Wills MR Carmichael AJ Mynard K Jin X Weekes MP Plachter B Sissons JG The human cytotoxic T-lymphocyte (CTL) response to cytomegalovirus is dominated by structural protein pp65: frequency, specificity, and T-cell receptor usage of pp65-specific CTL J Virol 1996 70 7569 7579 10.1128/jvi.70.11.7569-7579.1996 PMC190825 8892876

[B51] Kuzushima K Hayashi N Kimura H Tsurumi T Efficient identification of HLA-A*2402-restricted cytomegalovirus-specific CD8(+) T-cell epitopes by a computer algorithm and an enzyme-linked immunospot assay Blood 2001 98 1872 1881 10.1182/blood.v98.6.1872 11535524

