# Supplementary figures and images for: Functional impairment of Tax-specific but not cytomegalovirus-specific CD8+ T lymphocytes in a minor population of asymptomatic human T-cell leukemia virus type 1-carriers
Source: Retrovirology. 2011 Dec 7;8:100. doi: 10.1186/1742-4690-8-100 (PMC3261825; doi:10.1186/1742-4690-8-100)

Additional Figure 1

**A**

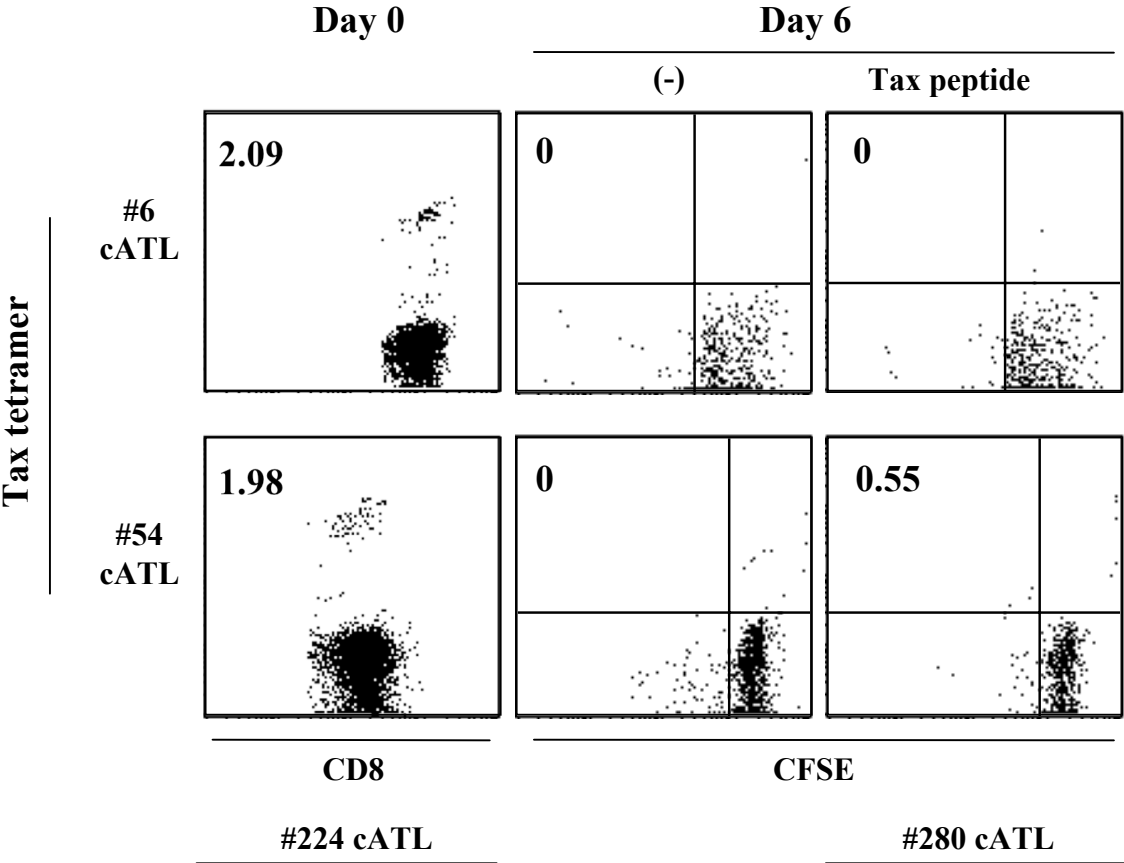

**B**

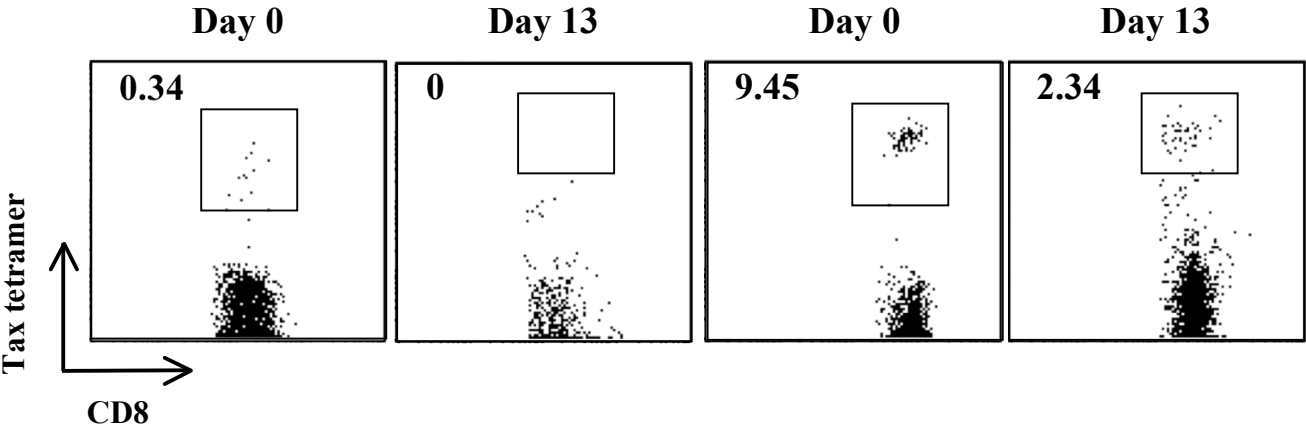

Supplement: Additional file 1 — Tax-specific CD8+ T-cells in cATL patients could not proliferate against Tax-peptide stimulation. (A) CFSE-labeled PBMCs were cultured with or without 100 nM Tax-peptide for 6 days. The number indicates the percentage of tetramer+ cells in CD8+ T cells (Day 0) or the percentage of dividing (CFSE low) cells in Tax-specific CD8+ T-cells (Day 6). In a cATL sample #54, CFSE-labeled PBMCs were cultured in the presence of mouse IgG for other experiment. (B) PBMCs (#224) and CCR4-depleted PBMCs (#280) were cultured for 13 days in the presence of 100 nM Tax-peptide. The number indicates the percentage of tetramer+ cells in CD8+ T-cells. [file 1742-4690-8-100-S1.PDF]
